# Global research trends in benign paroxysmal positional vertigo: a bibliometric analysis

**DOI:** 10.3389/fneur.2023.1204038

**Published:** 2023-06-02

**Authors:** Yuanjia Hu, Yang Lu, Shengyue Wang, Xiyu Quan, Yijia Ren, Kaiyi Rong, Sijia Pan, Xiaoyou Lu, Lei Chen, Chenghua Tian, Jianbo Lei

**Affiliations:** ^1^School of Medical Technology and Information Engineering, Zhejiang Chinese Medical University, Hangzhou, China; ^2^The Second School of Clinical Medicine, Zhejiang Chinese Medical University, Hangzhou, China; ^3^Clinical Research Center, The Affiliated Hospital of Southwest Medical University, Luzhou, China; ^4^School of Medical Informatics and Engineering, Southwest Medical University, Luzhou, China; ^5^Institute of Medical Technology, Peking University, Beijing, China

**Keywords:** BPPV, bibliometrics, hot topics, trends, vertigo

## Abstract

**Background:**

Benign paroxysmal positional vertigo is the most common disease in which vertigo is the main clinical manifestation, and it has become a global medical problem, affecting a wide range of areas and seriously affecting the quality of human life.

**Objective:**

This article presents an analysis of the current characteristics of BPPV-related research and summarizes the current hot topics and trends, with the goal of inspiring future research into the prevention and treatment of BPPV, thereby improving the differential diagnosis and prevention of peripheral vertigo.

**Methods:**

A bibliometric approach was used to collect 1,219 eligible studies on BPPV from four databases—PubMed, Embase, Scopus, and Web of Science—published between 1974 and 2022. The characteristics and status of the accumulated scientific output were processed using R and VOSviewer so that we could visualize any trends or hotspots.

**Results:**

The results showed a significant increase in the annual number of publications, with an average annual growth rate of 21.58%. A possible reason for the especially pronounced peak in 2021 was an increase in the prevalence of BPPV as a result of COVID-19. The new coronavirus became a focus of research in 2021. A total of 3,876 authors (of whom 1,097 were first authors) published articles in 307 different journals; 15.7% of the articles were published in *Acta Oto-Larygologica, Otology and Neurotology*, and *Frontiers in Neurology*. *Acta Oto-Laryngologica* was well ahead of the other journals in terms of growth rate and number of articles published. American scholars generated the largest number of articles overall, and the USA was involved in the greatest number of international collaborations, followed by Italy and China. The themes of the research centered around three topics, namely the treatment of BPPV, its influencing factors, and diagnosis.

**Conclusions:**

There has been a major increase in BPPV-related research over the last 50 years, leading to an increase in related articles and rapid development of the field. Key directions for future research include the improvement of individualized treatment for residual symptoms after initial treatment of BPPV among the elderly; effective control of comorbidities such as osteoporosis; and secondary inner ear disease, such as Ménière's disease.

## 1. Introduction

Benign paroxysmal positional vertigo (BPPV) is the most common form of self-limiting vestibular peripheral vertigo. It is closely associated with postural changes and accounts for 17% to 42% of patients with vertigo (1). Studies have shown that the rate of BPPV is 10.7 to 64/100,000 (2), accounting for approximately 25% of all clinical types of vertigo and 60% of peripheral vertigo (3). BPPV can involve the unilateral or bilateral semicircular canals (4) and is more common in the posterior and horizontal semicircular canals. It occurs in the posterior semicircular canal in 60–90% of cases, and in the horizontal semicircular canal in 5–30% (5). The anterior semicircular canal is rarely involved (6). It is now thought that the incidence of BPPV in the horizontal semicircular canal is underestimated. Despite its high prevalence, surprisingly little is known about the mechanisms underlying this disease and the health conditions associated with it (7).

Patients with BPPV often suffer from paroxysmal positional vertigo and nystagmus during specific movements, in addition to dizziness, nausea, imbalance, and impaired standing and walking. This can cause serious problems. Data from a study in Taiwan suggest that patients with BPPV have an increased risk of fracture for up to 12 years after diagnosis, with a particularly significant increase in the risk of spine, rib, and pelvis fractures. Such injuries can limit patients' movements and activity, thereby elevating the risk of other health problems (8). Beyond this, Joel Abbott et al. (9) found that more than half of patients admitted to the hospital for falls were positive on the Dix–Hallpike maneuver test and that patients with BPPV in the UK were at substantially increased risk of falls. In a US study, BPPV patients showed impaired static and dynamic balance, particularly when they were deprived of visual input and their proprioceptive input was altered, and most had difficulty balancing while standing on one leg with their eyes closed (10). It has even been shown that BPPV is associated with risk of ischemic strokes (11).

Unfortunately, there is a worrying lack of awareness of this disease, and diagnosis is often delayed. BPPV has also been found to be associated with a number of psychiatric symptoms (12), and some studies have shown that patients with BPPV may also suffer from anxiety, insomnia, social dysfunction, and even severe depression, with female patients potentially exhibiting more intense psychiatric abnormalities (13). Overall, these problems constitute a potentially huge burden upon patients and society, but there is no deep understanding of the harms and risks.

In this article, we seek to provide a complete and detailed description of the current state of the art and notable trends in BPPV research by using bibliometric methods to analyze relevant publications. As well as identifying important trends, we examine the geographical distribution of the work; describe the principal scholars, institutions, and journals in the field and the relationships between them; explore the strengths and limitations of current research; provide suggestions for further studies; and give detailed and comprehensive references.

## 2. Materials and methods

### 2.1. Data sources and search strategies

**Figure 1:**
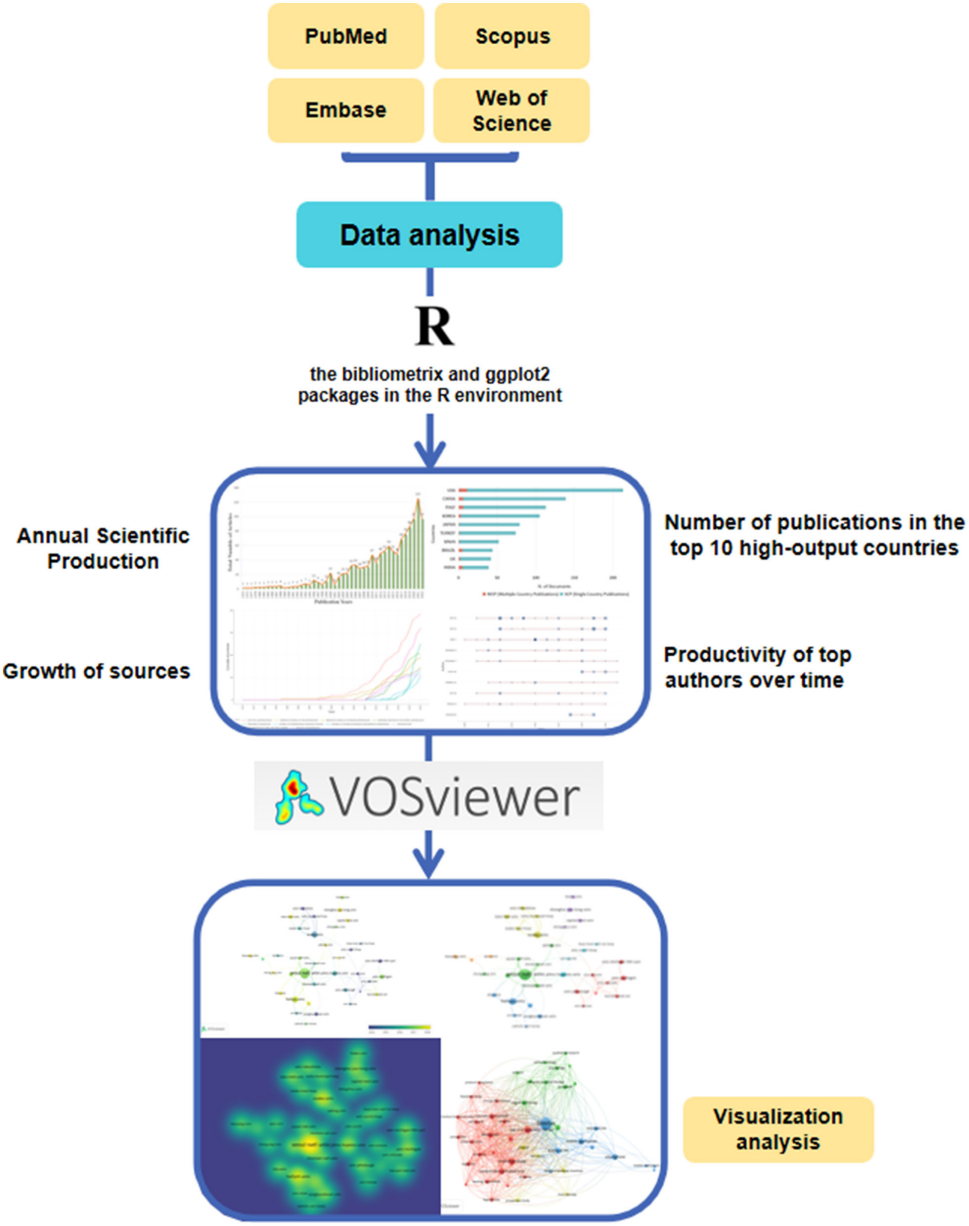
Principal methods and overview of the analyses conducted.

For completeness, a total of four databases in different fields were searched (14, 15). In the domain of medicine, PubMed, Embase, and Scopus were selected. For general science, the WoS core collection was selected. Search formulae were constructed with reference to established schemes and the search timeframe was up to October 2022. The search queries and strategies used for the different databases are listed in Supplementary Table 1. The main research methods and findings presented in this article are shown in Figure 1.

### 2.2. Inclusion and exclusion criteria

**Figure 2:**
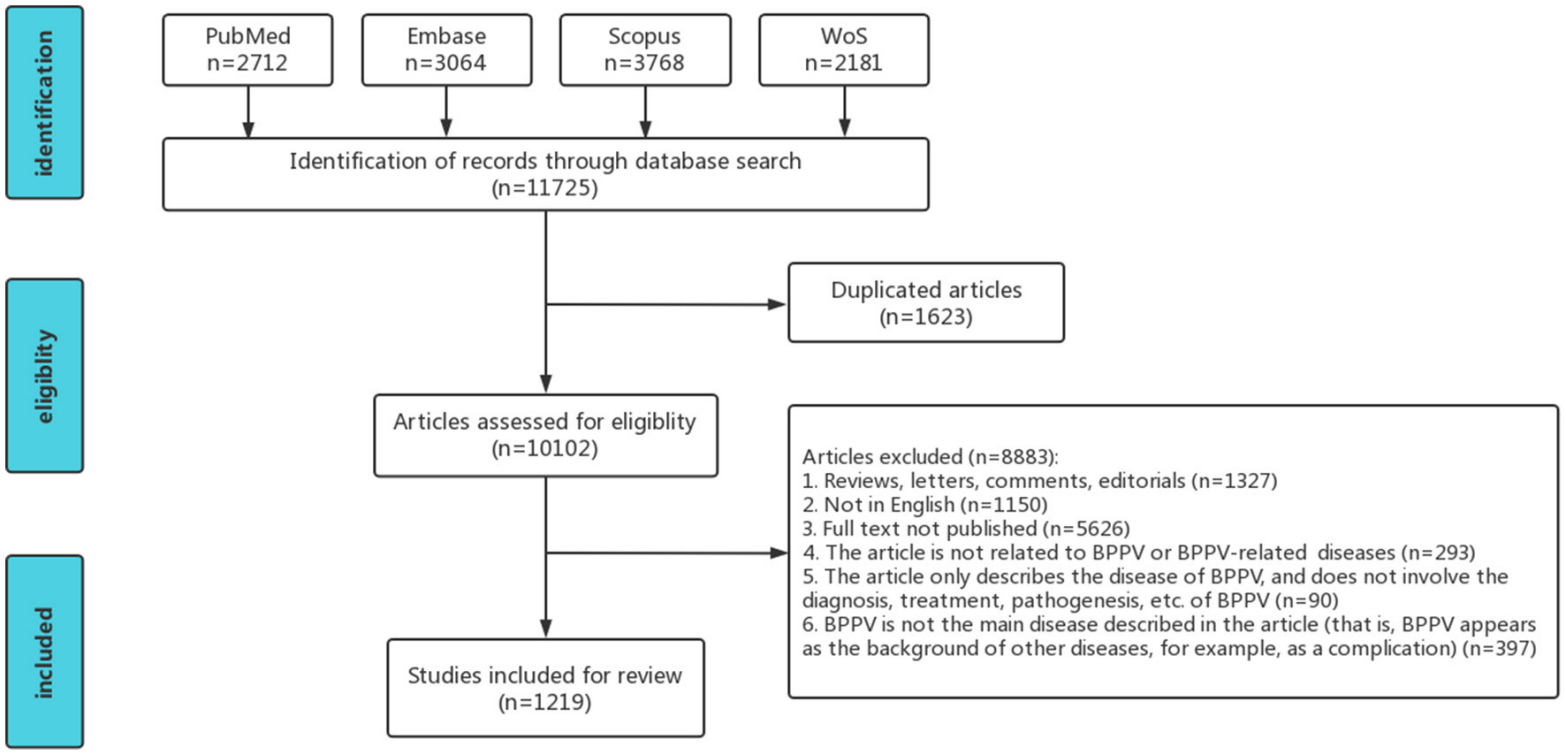
Literature screening flowchart.

As of October 2022, a total of 11,725 valid documents were retrieved from the four databases (2,712 from PubMed, 3,064 from Embase, 3,768 from Scopus, and 2,181 from WoS). Two trained postgraduate students with a medical background (audiology and clinical medicine) screened the retrieved literature according to specific inclusion and exclusion criteria (κ = 0.77). This resulted in a total of 1,219 titles being included, as illustrated in the screening flowchart in Figure 2.

### 2.3. Data analysis

The articles that met the relevant criteria having been collated, all bibliographic information was extracted from them. This included the title, abstract, author, author's affiliation address, author's nationality, publication date, publication name, and keywords. The literature was analyzed using the Bibliometrix package in R (16), ggplot2 (17) for statistical plotting, and VOSviewer (18) for network visualization.

## 3. Results

### 3.1. Analysis of publication trends

As of October 2022, a total of 3,876 authors had published 1,219 relevant articles in 307 different journals, including 1,097 first authors, with an average of 0.314 articles per author.

#### 3.1.1. General trends in the number of publications per year

**Figure 3:**
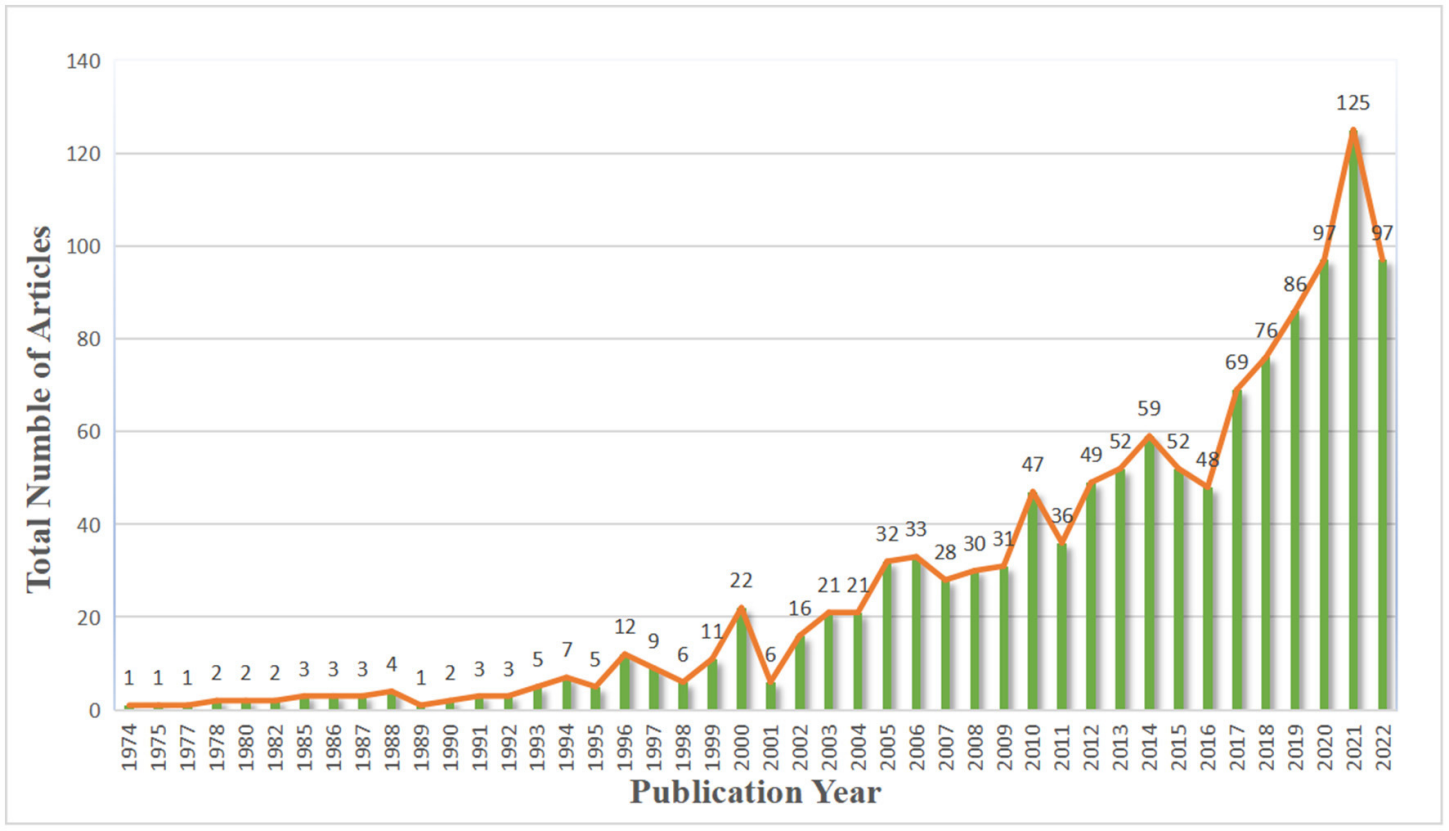
Annual scientific production.

Across the 1,219 articles that met the inclusion criteria, there was a notable year-on-year upward trend in publication rate from 1974 to 2022, with an average annual growth rate of 21.58%, peaking in 2021. A possible reason for the peak in 2021 is that COVID-19 caused an increase in the prevalence of BPPV. This is reinforced by the fact that several researchers began to focus on the new coronavirus in 2021, while no such articles were published in 2019 or 2020. A recent study noted a 183% increase in outpatient consultations for BPPV in 2020, showing there was an increase in the prevalence of BPPV during the period of the COVID pandemic (19). The number of relevant publications decreased slightly in 2022, but the overall level remained high, as shown in Figure 3. These results illustrate an increasing degree of concern with this topic, indicating an emerging need for more research.

#### 3.1.2. Analysis of journals of publication

**Figure 4:**
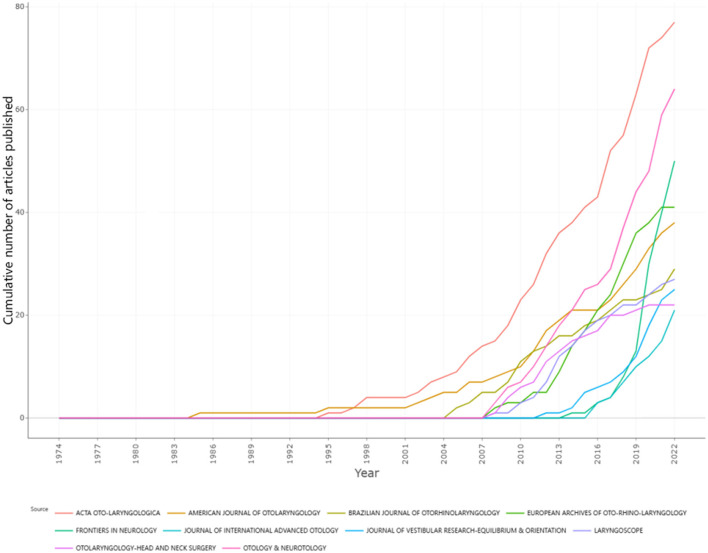
Comparison of the annual volume of relevant articles published in the core publications.

According to Bradford's law of scattering (20), the set of all publications operating within a field at a given time can be divided into those falling into the core, related, and peripheral zones, according to the number of articles carried. The ratio of the number of publications in the three zones is 1:*a*:*a*^2^, with *a* in this case approximating to 5 and the number of core publications accounting for approximately 1/31 of the total number. The core publications were calculated to be approximately the top 10. The articles published by these 10 journals accounted for 32.7% of all the relevant articles, as shown in Table 1. By plotting and comparing the annual trends in the number of core publications (see Figure 4), the journal *Acta Oto-Laryngologica* was found to be ahead of the other journals in terms of growth rate and the number of articles published. *Otology and Neurotology* and *Frontiers in Neurology* followed closely, but the remaining publications did not differ greatly in terms of the number of articles published.

**Table 1:** Ranking of core publications, JCR classification, and evaluation index.

**Rank**	**Source**	**Articles**	**JCR categories**	**2021 JIF**
1	Acta oto-laryngologica	77	Otorhinolaryngology	1.698
2	Otology and neurotology	64	Clinical neurology and otorhinolaryngology	2.619
3	Frontiers in neurology	50	Clinical neurology and neurosciences	4.086
4	European archives of oto-rhino-laryngology	42	Otorhinolaryngology	3.236
5	American journal of otolaryngology	38	Otorhinolaryngology	2.873
6	Brazilian journal of otorhinolaryngology	29	Otorhinolaryngology	2.474
7	Laryngoscope	27	Medicine, research and experimental and otorhinolaryngology	2.97
8	Auris nasus larynx	25	Otorhinolaryngology	2.119
9	Journal of vestibular research—equilibrium and orientation	25	Neurosciences and otorhinolaryngology	2.354
10	Otolaryngology–head and neck surgery	22	Otorhinolaryngology and surgery	5.591

### 3.2. Author analysis

The selected body of literature encompassed 3,876 authors. According to Lotka's law (21), which states that authors with more than 0.749 times the square root of the number of papers published by the most prolific scientists are high-producing authors, authors with more than four published works were defined here as high-producing authors. These accounted for 3.3% of all authors. For the purposes of illustration, the top 10 authors in terms of number of articles published are shown in Table 2.

**Table 2:** Top 10 authors in terms of number of articles published.

**Rank by number of articles**	**Author name**	**Total articles**
1	Kim JS	21
2	Kim HJ	18
3	Imai T	16
4	Inohara H	14
5	Kitahara T	11
6	Choi HG	10
7	Kerber KA	10
8	Lee SH	10
9	Takeda N	10
10	Bojrab DI	9

The number of works published by the top 10 authors shown in Table 2 includes those published as a co-author. To better understand the influential authors in this field, we also ranked the authors by number of works published as first author, as shown in Table 3. By comparing Tables 2, 3, it can be observed that two authors, IMAI T and KERBER KA, appear in both tables, indicating that these two authors may have made particularly extensive contributions to the field.

**Table 3:** Top 10 authors in terms of number of articles published as first author.

**Rank by number of articles**	**Author name**	**Total articles**
1	Imai T	9
2	Norre ME	9
3	Kerber KA	8
4	Pollak L	8
5	Lopez-Escamez JA	7
6	Balatsouras DG	6
7	Gacek RR	6
8	Korres S	6
9	Luryi AL	6
10	Ichijo H	5
11	Martellucci S	5
12	Parnes LS	5

#### 3.2.1. Analysis of publication trends among highly productive authors

**Figure 5:**
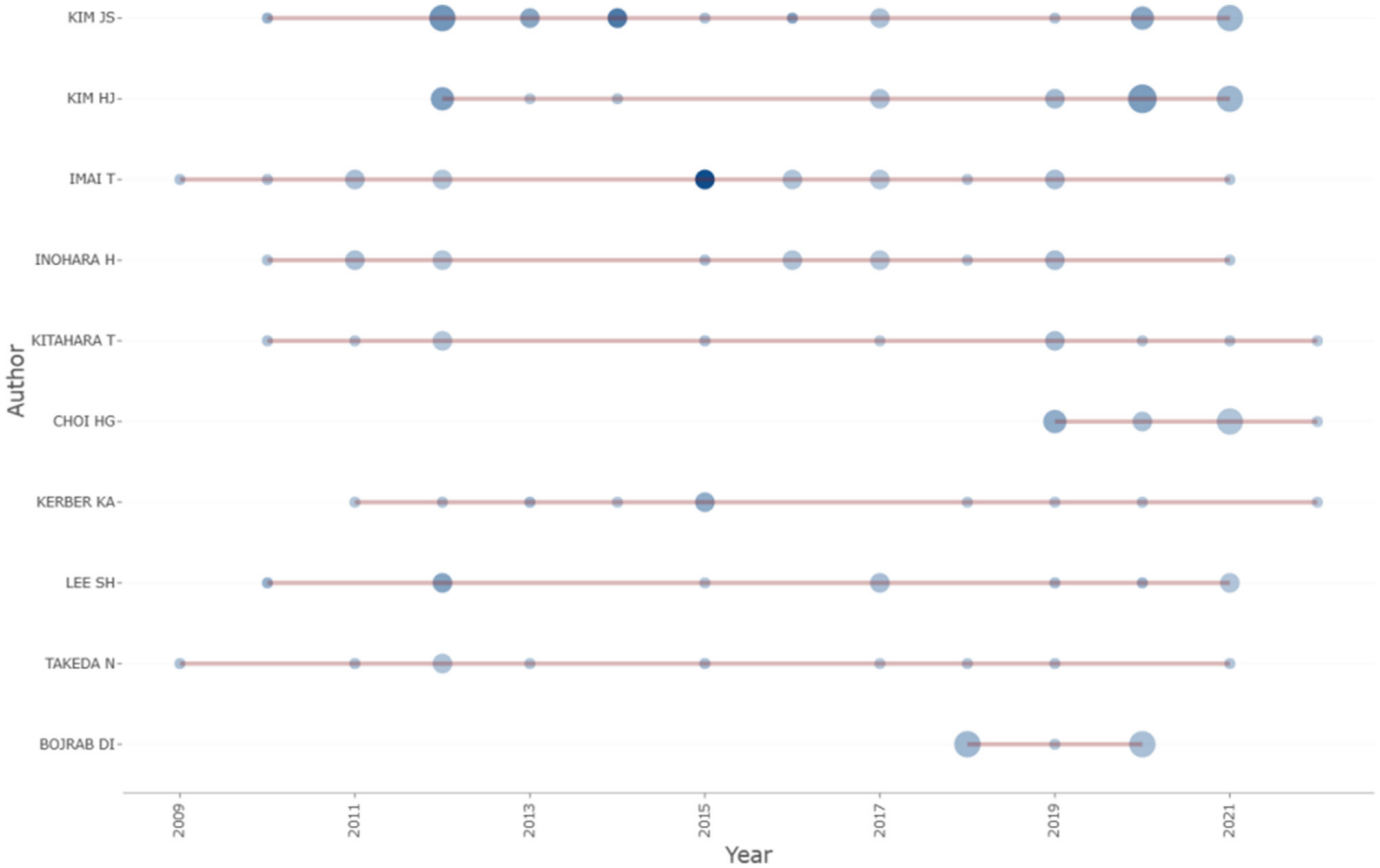
Productivity of top authors over time.

An annual publication chart for the top 10 authors, based on their annual publication data and annual citation frequency, is shown in Figure 5. The size of each circle represents the number of works published in the corresponding year, and the shade of blue represents citation frequency. Seven of the 10 authors started their research in the field after 2010, and two of them started their research after 2018, with stable output every year thereafter.

#### 3.2.2. Country analysis

**Figure 6:**
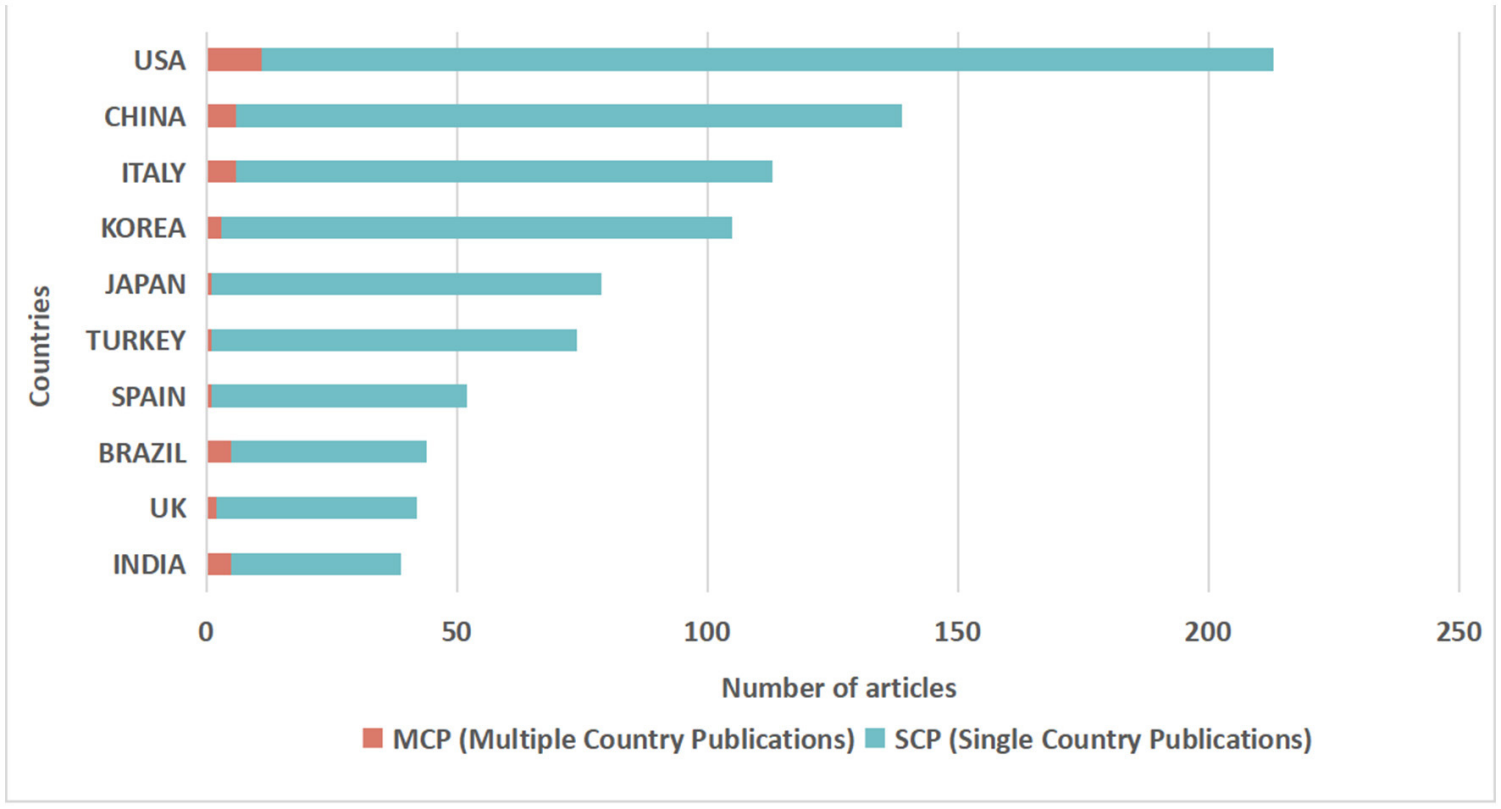
Articles by country of the corresponding author.

The 3,876 corresponding authors came from 40 countries altogether. The top 10 countries are shown in Figure 6. American authors published the largest number of articles in the field (213), followed by authors from China (139) and Italy (113). In terms of articles involving international collaboration, the USA was represented by the largest number of authors, followed by Italy and China, although this field has a relatively low rate of inter-country collaboration overall.

#### 3.2.3. Affiliation analysis

**Figure 7:**
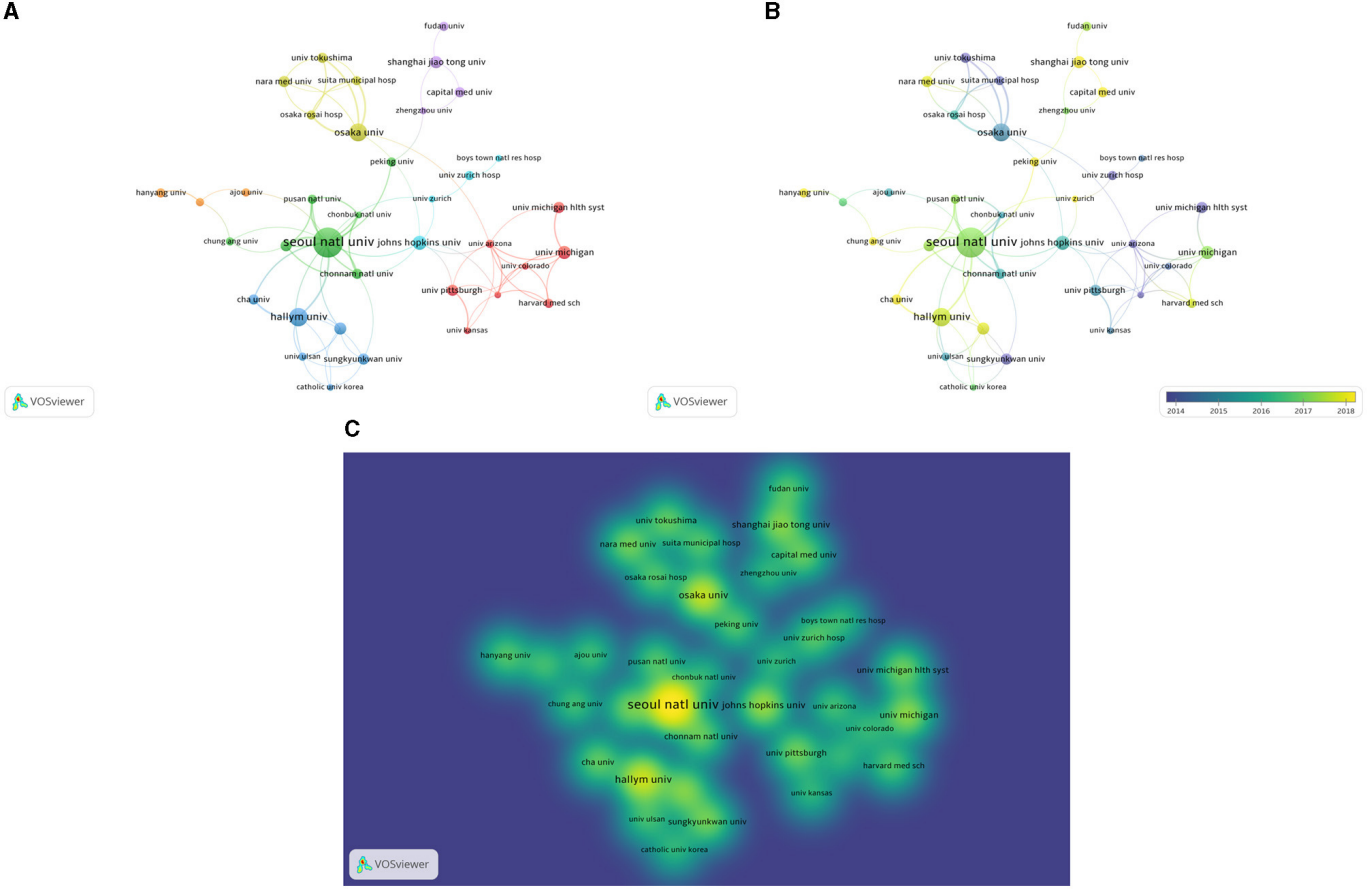
**(A)** Research institution-based collaborative networks. **(B)** Research publications by institution over time. **(C)** Density of publications by research institution.

The first authors across all the articles came from 1,000 research institutions, with Seoul National University, Osaka University, and Hallym University being the three most frequently represented institutions, followed by Shanghai Jiao Tong University and Johns Hopkins University. Authors from Seoul National University published a total of 31 relevant articles between 1974 and 2022. The co-authorship network between institutions is shown in Figure 7A with the green cluster representing the collaborative network led by Seoul National University, and the purple cluster above it representing the collaborative network dominated by Chinese research institutions, such as Shanghai Jiao Tong University and Fudan University. There was some collaboration between the clusters, but this is still in need of strengthening. Figure 7B presents a visual representation of research publications from each institution over time, while the more prominent colors in Figure 7C capture the relatively high volume of publications from Seoul National University, Osaka University, and Hallym University.

### 3.3. Analysis of topics published on

The topics of works published in the field were analyzed from three perspectives. First, high-frequency keywords were extracted from the abstracts of the selected articles. The relationships between these keywords were analyzed by establishing a high-frequency keyword co-occurrence network, and the main research topics were identified from the clustering results. Second, the thematic mapping technique proposed by Cobo et al. in 2011 was used to cluster themes according to their density and centrality to reveal any hotspots in the current body of research (22). Third, the evolution of various trends in the topics was analyzed over time. The top 40 subject-related keywords with the highest frequencies are shown in Supplementary Table 2.

#### 3.3.1. VOSviewer keyword clustering analysis

**Figure 8:**
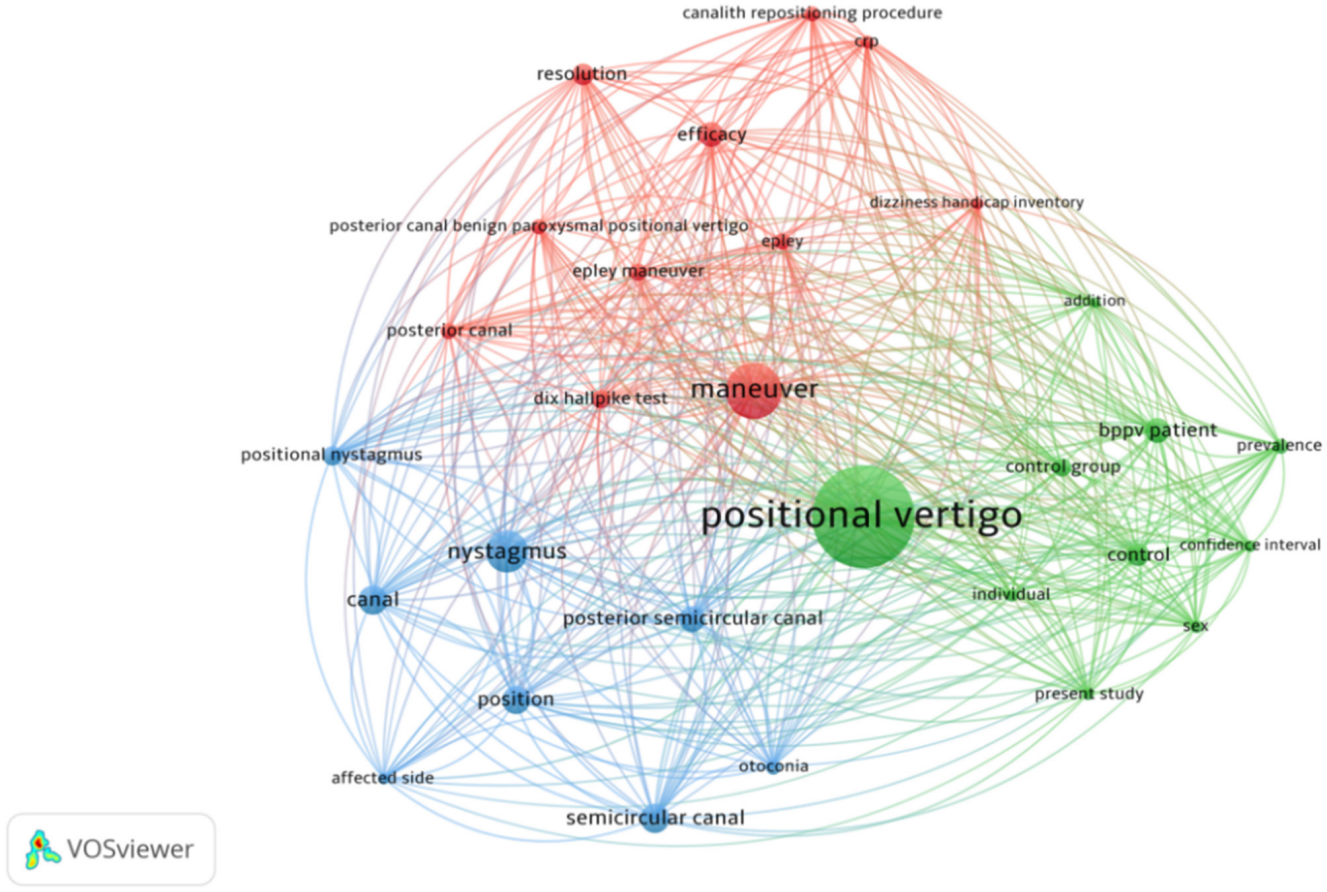
Keyword co-occurrence network.

As noted, a visual analysis of the co-occurrence networks for high-frequency keywords was undertaken. First, the abstracts from all of the chosen articles were divided, deactivated, and lexically normalized using Python to obtain all of the relevant keywords in the studies. A high-frequency keyword co-occurrence network was then produced using VOSviewer, as shown in Figure 8. It can be seen that the clustering analysis divided all the keywords into three main categories. The red cluster relates to treatment and diagnostic methods associated with the theme of 'maneuver'. The most frequently occurring terms were “Epley maneuver,” “Dix–Hallpike test,” and other specific treatments or diagnostic methods for otoliths. The blue cluster captures basic concepts such as “otoconia” and “posterior semicircular canal,” as well as clinical manifestations of otoliths, such as “nystagmus”. Finally, the green cluster represents research methods. Here, the high-frequency terms included “controlled study” and “control group,” as well as terms relating to potential influences on otoliths, such as “sex” and “prevalence”.

#### 3.3.2. Thematic map analysis

The thematic map method first proposed by Cobo et al. (22) uses the quadrants in a thematic map to analyze the importance of a topic and the extent to which it might be considered 'hot'. Themes in the first quadrant (very specialized/niche themes) are well-developed but less important; those in the second quadrant (motor themes) are well-developed and essential; the themes in the third quadrant (emerging or disappearing themes) are not well-developed and are of less concern; and those in the fourth quadrant (basic themes) are not well-developed but are necessary and generally relate to basic concepts.

**Figure 9:**
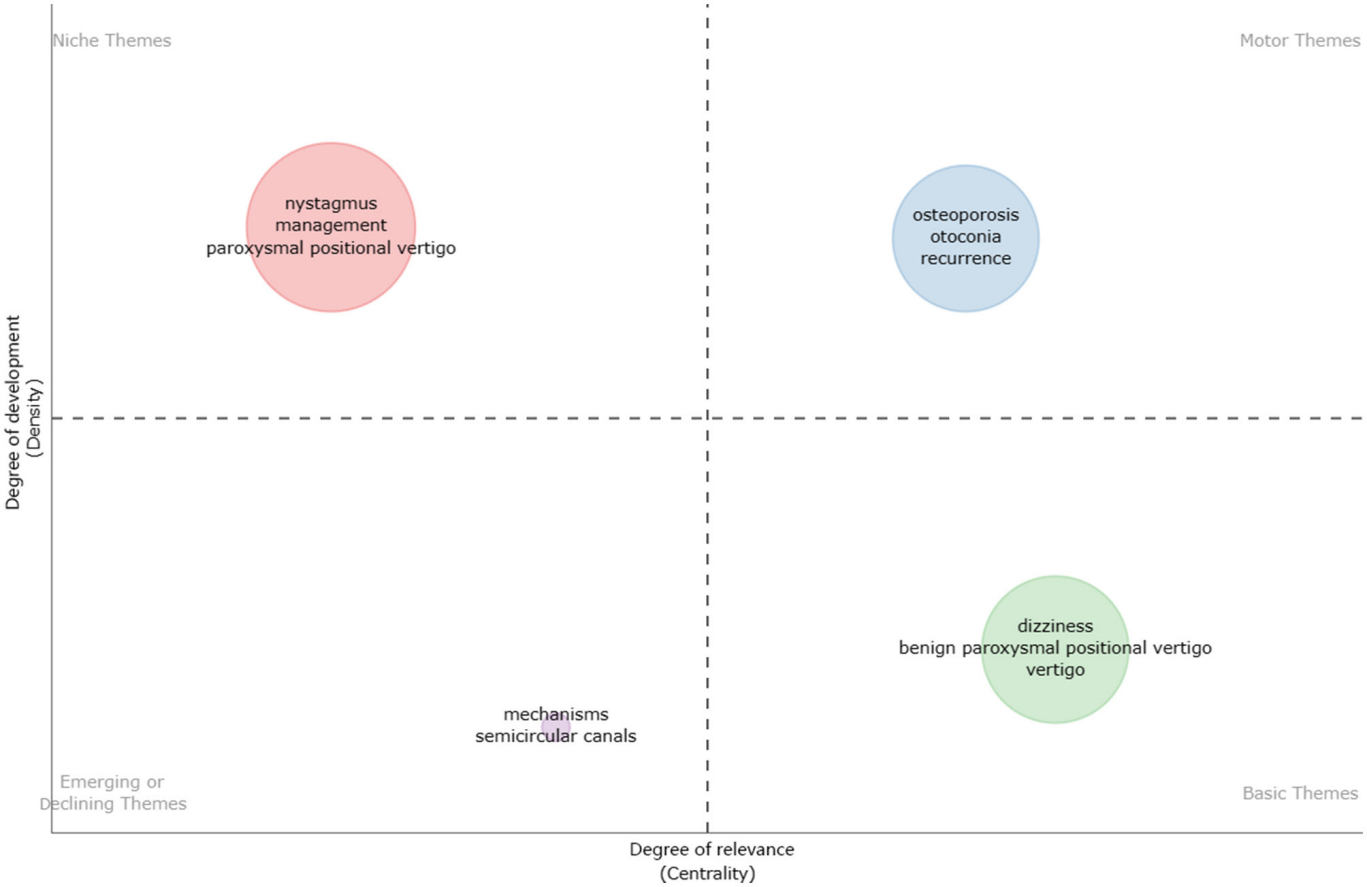
Thematic map of the domain.

By calculating the density and centrality of the existing clustered co-word matrix and visualizing each of the three categories in a two-dimensional coordinate system (see Figure 9), it was found that there was a predominance of terms in the first quadrant, such as “nystagmus” and other clinical manifestations. These are well-developed concepts that are now being phased out. In the second quadrant, the association between “osteoporosis” and “otolithiasis” and the recurrence of “otolithiasis” represent important and well-researched topics in the field. In the third quadrant, the exact pathogenesis of “otolithiasis” is not well developed. For the fourth quadrant, terms such as “vertigo” predominate and represent fundamental concepts in the field.

#### 3.3.3. Sankey diagram analysis

**Figure 10:**
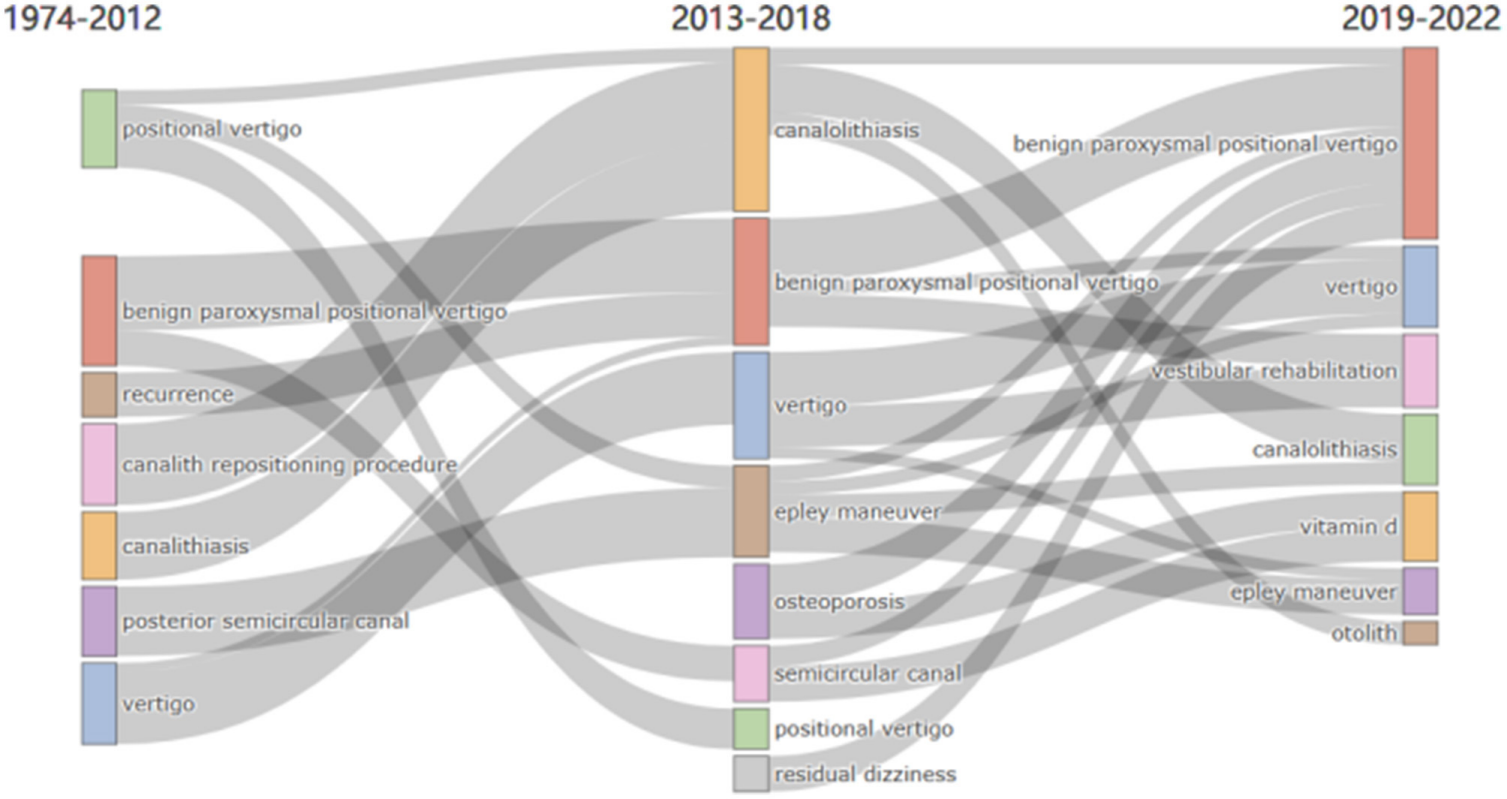
Sankey diagram showing how certain research themes have evolved.

A thematic evolution analysis was carried out, based on the co-word network analysis and clustering but incorporating a time dimension, so as to analyze the evolution of the research themes occurring over the selected period of 1974 to 2022. This analysis was then used to create a Sankey diagram, shown in Figure 10. It can be seen that some themes have changed over this period, but others have remained, with some researchers still concentrating on basic concepts such as the “posterior semicircular canal” and “vertigo.” It can also be seen that the concept of “benign paroxysmal positional vertigo” was already well-established and widely followed at the beginning of this period. Between 2013 and 2018, the pathogenesis of BPPV (e.g., “canalolithiasis”) and canalith repositioning maneuvers (e.g., the “Epley maneuver”) began to receive attention. From 2019 to 2022, this research shifted from a basic interest to potential applications. During this time, the concept of “vestibular rehabilitation” emerged and factors such as vitamin D began to be studied in relation to BPPV. By 2022, “Epley repositioning” had become a major research focus, so it seems likely that research regarding the pathogenesis of BPPV will be gradually expanded and extended over the next 2 years.

## 4. Discussion

### 4.1. Analysis of current publishing characteristics and trends

The number of papers published in this field has risen each year, with rapid growth after 2005 and a peak in the number of annual publications occurring in 2021. The journals in which the articles are being published are of moderate quality, but the acceptance rate for articles in the field by top medical journals is poor. In total, 64% of the selected papers were found in the WoS core repository, and the average impact factor of the journals they appeared in was 3.382, with a range of 0.147 to 19.334 (the *Journal of Trauma Injury Infection and Critical Care*). Currently, the principal journals that have published BPPV-related research are *Acta Oto-Laryngologica, Otology and Neurotology*, and *Frontiers in Neurology*, where a total of 191 articles (77, 64, and 50, respectively), or 15.7% of the total (141/1,219), had appeared as of October 2022. However, no relevant papers were published in NEJM or *The Lancet*, and only nine had appeared in JAMA and the BMJ, accounting for just 0.74% of the total.

The highest annual output for the top-producing authors was concentrated in 2021. Analysis revealed that the reason for this may be related to an increased prevalence of BPPV due to COVID-19. In accordance with Lotka's law, 128 of the most productive authors in the field were identified, all of whom have had a stable level of output over the last 3 years. This suggests that the field is growing at a rapid and stable rate. The most prolific authors are based in the USA, China, and Italy, with relatively few in other countries. Overall, 442 articles were published by American, Chinese, and Italian authors, accounting for 36.3% of all articles.

### 4.2. Hotspot analysis and likely future research directions

A thematic classification of the 1,219 selected papers revealed that 493 of them focused on the treatment of BPPV, 314 on the factors influencing its development, 264 on its diagnosis, and 86 on the hazards associated with BPPV. However, the prevention and pathogenesis of BPPV have been less studied, with a total of just 62 relevant publications taking an interest in this. Given this evidence, current research in this field appears to be primarily focused on the treatment of BPPV and the factors influencing it, rather than prevention of the disease.

#### 4.2.1. Diagnosis

BPPV is one of the most common peripheral vestibular disorders and is characterized by transient episodes of vertigo induced by head movements (23). The 2017 American Academy of Otolaryngology-Head and Neck Surgery Foundation (AAO-HNSF) guidelines on the clinical diagnosis of BPPV clearly state that the Dix-Hallpike test is the gold standard for the diagnosis of posterior hemiplegia BPPV. The Dix–Hallpike test specifically seeks to identify posterior canal otolithiasis and works by stimulating the posterior canal to induce nystagmus and vertigo when examining patients with suspected posterior canal BPPV (24). The side-lying test is considered by Cohen et al. to be a valid alternative to the Dix–Hallpike test and is more suitable for patients with limited mobility or who are unable to complete the Dix–Hallpike test (25). The supine roll test specifically looks for horizontal canal otolithiasis. The Dix–Hallpike test and the supine roll test are still considered to be the gold standard for BPPV diagnosis, while the caloric test forms the basis of all vestibular function tests, which are thought to help in the etiological analysis of BPPV. Some investigators have also found that the clinical use of blood biomarkers may be useful for BBPV diagnosis (26). Careful analysis is required in the diagnosis of BPPV, and, if necessary, nystagmus can be visualized using assistive devices such as Frenzel glasses or videonystagmography (2). These help with the identification of positional nystagmus, especially when it is weak or of short duration. Vestibular function tests can also be performed to assess vestibular function and, if necessary, provide corroboration of the diagnosis.

#### 4.2.2. Treatment

The treatment of BPPV has been the focus of attention in otology for more than 30 years, with repositioning maneuvers still being among the most effective methods of treatment for it. Depending on the type of BPPV, different kinds of repositioning maneuver may be used to facilitate the return of otolith fragments to the utricle (5). The Epley maneuver (27) is generally used to treat posterior canal otolithiasis; this is based on the theory of canalolithiasis, with the therapeutic goal being to move otolith fragments from the posterior canal to the utricle via gravity as a result of postural changes. This should eliminate the symptoms of vertigo. The Epley maneuver is now considered one of the most effective treatments for posterior canal otolithiasis. The Barbecue maneuver, also known as the Lempert maneuver, is commonly used to treat horizontal canal otolithiasis. It was introduced by Lempert et al. (28). Superior canal otolithiasis is believed to be rare in clinical practice, but a suitable treatment for the few cases that may arise is the Yacovino maneuver (29). Canalith repositioning maneuvers for BPPV are very effective, provide rapid relief, and prevent unnecessary investigations.

#### 4.2.3. Influencing factors

The factors associated with the development of BPPV have been another focus of study. According to the bulk of studies presented in the selected articles, the influencing factors can be divided into four categories: those that can cause BPPV to recur; those that can induce BPPV; factors that can aggravate the symptoms of BPPV; and those that may suppress its symptoms. The categories with the largest numbers of studies here were those covering factors that can induce BPPV and those that may cause it to recur; factors falling into these categories featured in 156 (60.5%) and 66 (25.6%) of the articles, respectively, for a total of 86.1%.

As noted, the majority of factor-related studies focus on factors that can induce BPPV. Some scholars (30, 31) have found that a lack of vitamin D may have this effect. Others (32, 33) have proved that traumatic brain injury (TBI) is related to the onset of BPPV and that BPPV after TBI may be more difficult to treat than idiopathic BPPV. Swimming has also been shown to be one of the precursors of BPPV (34). Rapid head movement during swimming can cause otoliths to fall off into the semicircular canal. Aging (35) and physical fatigue (36) also slightly increase the risk of BPPV. A study by Barber et al. (37) noted that repair of superior canal dehiscence (SCD) usually resolves preoperative auditory and vestibular symptoms, but many patients develop dizziness postoperatively. Postoperative dizziness may be the result of a new occurrence of BPPV, so repairing superior canal dehiscence may even induce BPPV. In recent years, researchers have conducted a wider range of studies on the potential predisposing conditions for the development of BPPV, and an increasing number of studies have begun to look at secondary BPPV.

With regard to factors associated with the recurrence of BPPV, a study by Fan et al. (38) has suggested that high serum levels of otolin-1 increase the risk of the condition recurring. Studies have also shown that otolith dysfunction can increase the recurrence rate (39). Lower than normal bone mineral density (BMD) is thought to be another factor that can predispose people to a recurrence of BPPV (40), and upright hypotension may also be a contributing factor (41). At the same time, BPPV patients should pay attention to sleeping position, as an incorrect sleeping position can also lead to the recurrence of BPPV (42). Naturally, some studies have focused their attention on ways to reduce the likelihood of BPPV recurring. For example, along with other scholars, Hong et al. (43) and Yang et al. (44) have suggested that vitamin D supplements can help to reduce the probability of recurrence.

To identify potential future topics for articles in this field, we counted the most frequently occurring subject-related terms in recent years and found that some of the most common ones included “maneuver” and “vertigo.” Different types of otolithiasis require different types of maneuvers to treat them. In recent years, studies have started to focus on this and on how existing maneuvers might be improved. The statistical findings also revealed the newfound prevalence of several previously infrequent terms, such as “Ménière's disease,” “sudden deafness,” “osteoporosis,” and “Vitamin D.” This suggests that secondary BPPV caused by inner ear diseases, such as Ménière's disease and sudden deafness, is becoming a hotter topic in this field of research.

### 4.3. Limitations of current research and suggestions for future development

As mentioned above, current research on BPPV is mainly focused on diagnosis, treatments, and influencing factors, and there is a certain degree of overlap between the different studies, so there has been no significant theoretical breakthrough in recent years. In the future, researchers should explore how to go beyond the limitations of current thinking and conduct groundbreaking BPPV research from different perspectives (e.g., vertigo modalities, treatment of comorbidities, or non-pharmacologic rehabilitation) to discover new insights, especially in the prevention and pathogenesis of BPPV. This would bring deeper and more accurate theoretical support to clinicians and provide the underpinning for a much more profound understanding of the pathogenesis of the disease so that it can be treated from its root causes. This, in turn, would lead to better treatments and prevention methods for BPPV patients, so that the chance of the disease occurring can be reduced before its onset, thereby reducing the prevalence of BPPV.

## 5. Conclusion

The bibliometric study reported in this article has shown that BPPV-related research has been steadily increasing in popularity since 1974, with a general increase in the number of relevant publications, up until their peak in 2021. The USA is the main country producing output in this domain. The main journal publishing relevant papers is *Acta Oto-Laryngologica*, with most of the research over the past 5 years appearing in *Frontiers in Neurology, Acta Oto-Laryngologica*, and the *Journal of Vestibular Research—Equilibrium and Orientation*. In the future, secondary BPPV caused by inner ear diseases, such as Ménière's disease and sudden deafness, is likely to become a particular focus of research, while factors such as osteoporosis and vitamin D deficiency will be increasingly accepted as influencing the development of BPPV.

## Data availability statement

The raw data supporting the conclusions of this article will be made available by the authors, without undue reservation.

## Author contributions

Conception and design: YH. Administrative support and provision of study materials: CT and JL. Collection and assembly of data: YH, LC, YR, and XL. Data analysis and interpretation: YH, YL, KR, SP, SW, and XQ. Manuscript writing and final approval of manuscript: All authors.
