# Supplementary material for: Global research trends in benign paroxysmal positional vertigo: a bibliometric analysis
Source: Front Neurol. 2023 Jun 2;14:1204038. doi: 10.3389/fneur.2023.1204038 (PMC10272773; doi:10.3389/fneur.2023.1204038)
Supplement: Supplementary file 1 [file Table_1.DOCX]

Supplementary Material

Global research trends in benign paroxysmal positional vertigo: Bibliometric analysis

**Yuanjia Hu^1^, Yang Lu^1^, Shengyue Wang^1^, Xiyu Quan^1^, Yijia Ren^1^, Kaiyi Rong^2^, Sijia Pan^1^, Xiaoyou Lu^1^, Lei Chen^1^, Chenghua Tian^1^**^*^**, Jianbo Lei^3,4,5^**^*^

***Correspondence:**Chenghua Tian^1*^
Email address: [20071044@zcmu.edu.cn](mailto:20071044@zcmu.edu.cn)

Jianbo Lei^3,4,5*^
Email address: [jblei@hsc.pku.edu.cn](mailto:jblei@hsc.pku.edu.cn)

# Supplementary Tables

**Supplementary Table 1***. Query strategies and results*

| **Database** | **Query** | **Results** | **Date of Query** |
| --- | --- | --- | --- |
| Pubmed | ('Benign Paroxysmal Positional Vertigo'/exp OR Benign Paroxysmal Positional Vertigo) OR ('BPPV'/exp OR BPPV) | 2712 | 2022.10 |
| Embase | 'benign paroxysmal positional vertigo':ti,ab OR bppv:ti,ab | 3064 | 2022.10 |
| Scopus | TITLE-ABS-KEY (benign paroxysmal positional vertigo) OR TITLE-ABS-KEY(bppv) | 3768 | 2022.10 |
| WoS core collection | TS= ("Benign Paroxysmal Positional Vertigo" OR BPPV) | 2181 | 2022.10 |

**Supplementary Table 2.** *Top 40 most frequent subject headings*

| Keywords | Number of occurrences |
| --- | --- |
| Benign paroxysmal positional vertigo | 1567 |
| vertigo | 760 |
| Dizziness | 518 |
| Major clinical study | 496 |
| Controlled study | 496 |
| Nystagmus | 428 |
| Semicircular canal | 420 |
| Diagnosis | 219 |
| Body position | 216 |
| Meniere disease | 198 |
| Vestibular disorder | 194 |
| Retrospective study | 190 |
| Prospective study | 179 |
| Case report | 174 |
| patient positioning | 171 |
| Therapy | 152 |
| Otolith | 134 |
| Recurrent disease | 126 |
| Dix Hallpike test | 114 |
| Vestibular neuronitis | 107 |
| Prevalence | 105 |
| Epley maneuver | 103 |
| Complications | 101 |
| Semicircular canals | 101 |
| Nuclear magnetic resonance imaging | 97 |
| Vestibular test | 97 |
| Canalolithiasis | 94 |
| Positional vertigo | 92 |
| Randomized controlled trial | 85 |
| Migraine | 83 |
| Caloric vestibular test | 72 |
| Comorbidity | 66 |
| Vestibular evoked myogenic potential | 64 |
| Osteoporosis | 63 |
| Cupulolithiasis | 61 |
| Vitamin D | 61 |
| Prognosis | 57 |
| canalith repositioning procedure | 57 |
| Barbecue maneuver | 17 |
| Lempert maneuver | 8 |
| Roll test | 7 |
